# Antinociceptive and Anti-Inflammatory Effects of Syringic Acid: *In Vivo* Evidence and *In Silico* Mechanistic Insights

**DOI:** 10.1021/acsomega.6c00914

**Published:** 2026-09-10

**Authors:** Şeyda Yön, Cevşen Yazıcı Şenocak, Gizem Türkoğlu Sağlık, Ümmühan Kandemir, Mustafa Eser, Asaf Evrim Evren, Özgür Devrim Can, Ümide Demir Özkay

**Affiliations:** † Department of Pharmacology, Graduate School, Anadolu University, Eskişehir 26470, Türkiye; ‡ Department of Pharmacology, Faculty of Pharmacy, Anadolu University, Eskişehir 26470, Türkiye; § Department of Medical Pharmacology, Faculty of Medicine, Bilecik Şeyh Edebali University, Bilecik 11100, Türkiye; ∥ Department of Pharmaceutical Microbiology, Faculty of Pharmacy, 52944 Anadolu University, Eskişehir 26470, Türkiye; ⊥ Department of Pharmaceutical Chemistry, Faculty of Pharmacy, 52944 Anadolu University, Eskişehir 26470, Türkiye

## Abstract

Based on the previously reported neuroprotective, neurochemical, and behavioral effects of syringic acid, the present study was designed to investigate its antinociceptive and anti-inflammatory potential and to elucidate its possible mechanisms of action using *in vitro* and *in silico* methods. The antinociceptive effects of syringic acid were evaluated using the tail-clip, tail-immersion, hot-plate, formalin, and acetic acid-induced writhing tests in Balb/c mice, whereas its anti-inflammatory activity was assessed using the carrageenan-induced paw edema model in Sprague-Dawley rats. Motor performance was examined using the activity meter and Rota-rod tests. The potential ulcerogenic effect was also evaluated. Syringic acid (50 and 100 mg/kg, *p.o.*) did not significantly alter motor performance. It also did not alter nociceptive response parameters in the tail-clip, tail-immersion, or hot-plate tests. However, it significantly decreased writhing behavior in the writhing test and reduced paw-licking time during the second phase of the formalin test. Moreover, syringic acid attenuated carrageenan-induced paw edema. These findings indicate that syringic acid exerts peripheral antinociceptive and anti-inflammatory activities. In addition, acute administration of syringic acid did not induce gastric ulceration in mice. Enzyme inhibition studies showed that syringic acid inhibited cyclooxygenase-2 and 5-lipoxygenase at μM concentrations. *In silico* studies further revealed stable interactions between syringic acid and both enzymes.

## Graphical abstract

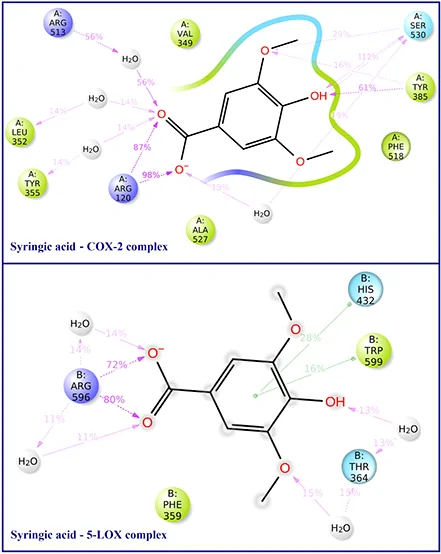

## 1 Introduction

Syringic acid, a phenolic compound, is present in various plant-based sources, including dates, grapes, olives, squash, acai palms, some spices, red wine, and honey.^1^ To date, syringic acid has been reported to exert antioxidant,^1^ antimicrobial,^2^ anticancer,^3^ antihyperglycemic,^4^ cardioprotective,^5^ hepatoprotective,^6^ renoprotective,^7^ and antisteatotic^8^ effects.

In the literature, several studies have investigated the central nervous system (CNS)-related activities of syringic acid. Previous studies have demonstrated that syringic acid exhibits antidepressant-like effects^9,10^ and ameliorates anxiety-like behaviors in animals with thioacetamide-induced hepatic encephalopathy.^11^ The beneficial effects of syringic acid on cognitive impairment have also been demonstrated in various experimental models, such as rats exposed to subchronic deltamethrin,^12,13^ streptozotocin-induced diabetic rats,^14^ and aluminum chloride-induced Alzheimer’s disease models.^15^ Moreover, syringic acid has been shown to improve motor impairments observed in various animal models.^11,12,14–17^

The literature also contains several studies reporting that syringic acid exhibits neuroprotective activity by preventing glutamate-induced neurotoxicity^9,10^ and inhibiting neurodegeneration caused by thioacetamide,^11^ aluminum chloride,^15^ 1-methyl-4-phenyl-1,2,3,6-tetrahydropyridine/probenecid (MPTP/p),^16,17^ deltamethrin,^12^ or surgical procedures.^18–20^ Some studies link the neuroprotective effects of this phenolic compound to its effects on inflammatory mediators.^11,15,16^ The suppressive effects of syringic acid on inflammatory processes have been supported in various experimental models, such as dimethyl nitrosamine-induced hepatic injury,^21^ high-fat and high-fructose diet-induced hyperglycemia,^22^ complete Freund’s adjuvant (CFA)-induced arthritis,^23^ and methyl cellosolve-induced hepatic and testicular inflammation.^24^

Although numerous studies have demonstrated the neuroprotective, neurochemical, and behavioral effects of syringic acid, reports on its acute antinociceptive and anti-inflammatory activities remain limited.^25,26^ The available evidence indicates the antinociceptive effects of syringic acid only against thermal nociceptive stimuli and anti-inflammatory activity in Wistar rats at doses of 25 and 50 mg/kg. However, several aspects of this phenolic compound, such as its time-dependent central antinociceptive effects against both thermal and mechanical nociceptive stimuli, peripheral antinociceptive potential, anti-inflammatory activity in another animal strain and at a higher dose, possible ulcerogenic effects on the gastric mucosa, as well as its inhibitory effects on the key molecular targets COX and 5-LOX, interactions with these enzymes, and the stability of the resulting complexes, have not yet been comprehensively investigated. Therefore, this study aims to assess the probable central and peripheral antinociceptive and anti-inflammatory effects of syringic acid using multiple validated *in vivo* models within a comprehensive experimental design and to provide insights into its possible mechanism of action through *in vitro* studies and *in silico* methods.

## 2 Materials and Methods

### 2.1 Chemicals

Syringic acid, diclofenac sodium, morphine sulfate, λ-carrageenan, acetic acid, formalin solution, and Tween 80 were acquired from Sigma (St. Louis, MO, USA), while serum physiologic was supplied by Osel (Beykoz, İstanbul, Türkiye).

### 2.2 Animals

This study was conducted with Balb/c mice (male, 30–35 g) and Sprague-Dawley rats (male, 250–300 g). Animals were kept in well-ventilated rooms with constant humidity, temperature (24 ± 1°C), and a 12-h light-12-h dark cycle. The animals were transferred to the laboratories at least 48 h prior to the tests and were allowed to adapt to the environmental conditions. Ethical approval for the study was obtained from the Local Ethical Committee on Animal Experimentation of Anadolu University, Eskişehir, Türkiye (Approval Date: May 11, 2022; Approval No.: 2022–17).

### 2.3 Administration of Drugs and Agents

Animals were randomly assigned to the experimental groups using the online tool QuickCalcs (GraphPad Software, San Diego, CA, USA). The researchers were blinded to group assignments during the experimental procedures, outcome assessments, and data analysis.

Syringic acid (≥95% purity) was dissolved completely in Tween 80 (1%, v/v) and freshly prepared just before the administrations. No color change or precipitation was observed in the prepared solutions. Syringic acid was administered to animals at doses of 50 and 100 mg/kg, in accordance with previous studies reporting pharmacological efficacy without toxicity. This phenolic compound was administered orally, as this route has been commonly used in previous *in vivo* studies^11,26^ and is considered clinically relevant. A single-dose protocol was used to assess the acute antinociceptive and anti-inflammatory effects of syringic acid. Animals in the control group received a solution containing Tween 80 (1%, v/v) orally.

Morphine (≥98% purity) (10 mg/kg, *i.p.*), a centrally acting analgesic, was used as a positive control in the tail-clip, tail-immersion, and hot-plate tests.^27^ For the formalin test, which evaluates both central and peripheral antinociceptive effects, morphine, known to inhibit both phases,^28,29^ was used for the validation of the test. Diclofenac sodium (≥98% purity) (20 mg/kg, *i.p.*) was preferred as the reference drug in the acetic acid-induced writhing and carrageenan-induced paw edema tests due to its well-established peripheral analgesic and anti-inflammatory effects, respectively.^30^ Morphine sulfate and diclofenac sodium were dissolved in physiological saline and freshly prepared just before the administrations.

All tests were performed 60 min following oral syringic acid administration.^27^

### 2.4 Motor Activity Tests

#### 2.4.1 Activity Meter Test

The possible effect of syringic acid on motor activities of the animals was assessed with an activity meter device (Commat, MayAMS02, Ankara, Türkiye). One hour after the oral administration of syringic acid or vehicle, each mouse was placed in the center of the apparatus, and its ambulatory activity, numbers of horizontal and vertical movements, and walking distance were recorded for 5 min. The device was cleaned using ethanol after each animal.^17^

#### 2.4.2 Rota-Rod Test

The possible effect of syringic acid on motor coordination of the animals was evaluated using a Rota-rod apparatus (Ugo Basile, 47650, Varese, Italy). Before the experiments, the mice were trained for 3 days on a rod rotating at 16 revolutions per minute (rpm), and only mice that remained on the rod for longer than 180 s were included in the study. In the test stage, 60 min after the oral administration of syringic acid or vehicle, the mice were placed back on the rotating rod, and the latency to fall for each animal automatically recorded by the device.^27,31^

### 2.5 Nociceptive Tests

#### 2.5.1 Tail-Clip Test

In the tail-clip test, animals were exposed to a mechanical nociceptive stimulus. In this procedure, a metal artery clamp is attached to the tail of the animal, and the latency until the animal turns and bites the clamp was recorded using a stopwatch. An increase in the response latency was interpreted as an indicator of antinociceptive activity. Prior to the tests, a sensitivity assessment was performed, and only animals that responded to the mechanical painful stimulus within 10 s were included in the experiments. To avoid potential tissue damage, exposure to the nociceptive stimulus was limited to 10 s.^27,32^

#### 2.5.2 Tail-Immersion Test

In the tail-immersion test, animals are exposed to a thermal nociceptive stimulus. In this procedure, the distal 1–2 cm segment of the each animal’s tail was submerged in water maintained at 52 ± 1 °C, and the tail withdrawal latency was measured using a stopwatch. An increase in withdrawal latency is considered an indicator of antinociceptive activity. Before testing, a sensitivity assessment was performed, and only animals that responded to the thermal painful stimulus within 4 s were selected for the experiments. To prevent tail injury, the nociceptive stimulus was not applied for longer than 20 s.^28,31^

#### 2.5.3 Hot-Plate Test

The hot-plate test was carried out using a hot/cold plate apparatus (Ugo Basile, 35300, Varese, Italy) to evaluate thermal nociception. In this assay, mice were positioned on a hot plate maintained at 55 ± 1.0 °C, and the latency to the first occurrence of paw licking or jumping is recorded with a stopwatch. An increase in response latency was considered an indication of an antinociceptive effect. Sensitivity studies were conducted before testing, and experiments were performed only with animals that responded to the thermal painful stimulus within 15 s. To prevent paw injury, exposure to the nociceptive stimulus was restricted to 30 s.^27,33^

In all three tests, reaction latencies were measured before the treatments and at 30, 60, 90, 120, and 180 min after administrations. From the obtained latencies, the percentage of maximum possible effect (MPE %) was also calculated using the following formula:

$$MPE\%=\frac{Pos\mathrm{t‐}treatment\;latency-Baseline\;latency}{Cu\mathrm{t‐}off\;time-Baseline\;latency}\times 100$$

The MPE–time data obtained over 0–180 min were used to calculate the corresponding area under the curve (AUC).^31^

#### 2.5.4 Acetic Acid-Induced Writhing Test

In the acetic-acid-induced writhing test, a chemical nociceptive stimulus was applied to animals. In this test, 1 h after the oral administration of syringic acid or vehicle and 30 min after the *i.p.* administration of diclofenac, abdominal pain and writhing response were induced by administering a 0.6% acetic acid solution (0.1 mL for 10 g body weight) to mice via *i.p.* route. The writhing behavior, characterized by stretching of the hind limbs and abdominal constriction, was recorded for 10 min starting 5 min after acetic acid administration. A decrease in the writhing count was considered an indicator of antinociceptive activity.^27,34^

For this test, the percentage of antinociceptive activity was also determined according to the following equation:

$$Antinociceptive\;activity\%=\frac{Mean\;writhing\;count\left(control\right)-Mean\;writhing\;count\left(treatment\right)}{Mean\;writhing\;count\left(control\right)}\times 100$$

#### 2.5.5 Formalin Test

The formalin test, similar to the acetic acid-induced writhing test, is a nociceptive test in which a chemical stimulus is applied to animals. In this test, 1 h after the oral administration of syringic acid or vehicle and 30 min after the *i.p.* administration of morphine, formalin solution (2.5%, 20 μL) was injected subcutaneously (*s.c.*) into the right hind paw of each mouse. Paw licking duration was measured using a stopwatch during both the early (0–5 min) and late (15–30 min) phases following formalin administration. Reductions in paw-licking time was considered an indicator of antinociceptive activity.^35,36^

### 2.6 Inflammation Test

#### 2.6.1 Carrageenan-Induced Paw Edema Test

The anti-inflammatory effect of syringic acid was assessed in a carrageenan-induced paw edema model. In this assay, 1 h after the oral administration of syringic acid or vehicle and 30 min after the *i.p.* administration of diclofenac, 1% (w/v) λ-carrageenan solution (0.1 mL) was injected subplantarly into the right hind paw of each rat. Paw volume was measured using a plethysmometer device (Ugo Basile, 37240, Varese, Italy) before carrageenan injection and at 1, 2, 3, 4, and 24 h thereafter.^37^

Swelling ratio percentages were calculated using data acquired from this test. The formula used for this calculation is as follows:

$$Swelling\;ratio=\frac{Paw\;volume\;at\;each\;time\;point-the\;initial\;paw\;volume}{The\;initial\;paw\;volume}\times 100$$

### 2.7 Gastric Ulceration Studies

Gastric ulcerogenic activity potential of the acute syringic acid administration was assessed, as described earlier.^37,38^ After 12 h of fasting, the mice were euthanized and their stomachs were removed. Each stomach was opened along the greater curvature, rinsed thoroughly with saline, and stretched with pins on a board. Gastric mucosa was macroscopically evaluated under a dissecting microscope and scored on a scale ranging from 0 to 7. Scores were assigned as follows: 0, no visible lesion; 1, color alteration; 2, mild petechia; 3, one to three small lesions (≤1 mm in length); 4, one to three large lesions (≥1 mm in length); 5, large lesions (>1 mm in length); 6, several continuous linear lesions or small non-bleeding ulcers and 7, several massive bleeding or digested blood in the stomach.

### 2.8 Statistical Analysis

GraphPad Prism ver. 8.2.1 (GraphPad Software, San Diego, CA, USA) was used for statistical analyses. Graphs were generated and the area under the curves (MPE %-time) were determined with the same software. All data were tested for normality using Shapiro-Wilk test. Data obtained from the motor activity, acetic acid-induced writhing, formalin, and carrageenan-induced paw edema tests and AUC values were evaluated by one-way analysis of variance (ANOVA) with Tukey’s honestly significant difference (HSD) post hoc test. Data obtained from time-course nociceptive tests were analyzed using two-way repeated measures ANOVA with Tukey’s HSD post hoc test. In this analysis, the Greenhouse–Geisser correction was applied to adjust the degrees of freedom and reduce the risk of type I error. Effect sizes (η^2^ for one-way and η^2^p for two-way repeated measures ANOVA) were calculated.

### 2.9 *In Vitro* Enzyme-Based Assays

Enzymatic inhibitory activities against COX-1 (COX-1 Inhibitor Screening Kit, Catalog Number: ab204698), COX-2 (COX-2 Inhibitor Screening Kit, Catalog Number: ab283401), and 5-LOX (Lipoxygenase Inhibitor Screening Assay Kits, Catalog Numbers: 760700 and 60401) were determined using commercially available assay kits according to the manufacturer’s instructions. For each enzyme, all measurements were performed in quadruplicate. As in previous reports,^39,40^ the half-maximal inhibitory concentration (IC_50_) values were calculated based on the protocol and analysis methods provided by the supplier. As supplied in the kits, SC560, celecoxib, and nordihydroguaiaretic acid (NDGA) were used as reference molecules for COX-1, COX-2, and 5-LOX enzymes, respectively.

### 2.10 *In Silico* Studies

#### 2.10.1 Molecular Docking

Molecular modeling studies were run to define the binding modes of syringic acid in the active pockets of COX-1, COX-2, and 5-LOX enzymes. Their X-ray crystal structures (PDB IDs: 6COX and 6N2W) were retrieved from the Protein Data Bank server (www.pdb.org, accessed 03 April 2022).

The crystal structures of enzymes were built using the interface and were then submitted to the Protein Preparation Wizard protocol of the Schrödinger Suite 2020.^41^ The tautomeric forms and protonation states of syringic acid were generated using the LigPrep module.^42^ The most probable ionization states at physiological pH (7.4 ± 1.0) were assigned automatically, together with appropriate atom types. Bond orders were verified, and hydrogen atoms were added to obtain the final three-dimensional ligand structures for molecular docking and molecular dynamics simulations. The grid generation was formed using the Glide module,^43^ and docking runs were performed in standard precision docking mode.

#### 2.10.2 Molecular Dynamics Simulations

Molecular dynamics (MD) simulations have been used to understand the time-dependent stability of a ligand into an active site for the ligand-protein complexes.^44,45^ MD simulations for 100 ns were carried out to analyze the stability of the identified hits from the docking results.

Using Desmond application, interaction behavior was evaluated during MD simulation time.^46^ All systems were set up using “System Builder” in Maestro. The standard procedure was applied in System Builder and Molecular dynamics interface.^47^ After the neutralization of the system was achieved using Na^+^ and Cl^–^ ions, the system setup was done and then the molecular dynamic simulation was performed using the system complex.

## 3 Results

### 3.1 Pharmacology

#### 3.1.1 Findings of Motor Activity Tests

The effect of syringic acid (50 and 100 mg/kg) administrations on ambulatory ([F­(2,21) = 1.01, *p* = 0.3821, η^2^ = 0.08756]) (Figure 1A), horizontal ([F­(2,21) = 0.49, *p* = 0.6166, η^2^ = 0.04500]) (Figure 1B), vertical ([F­(2,21) = 0.71, *p* = 0.5021, η^2^ = 0.06351]) (Figure 1C) activity counts, as well as walking distance ([F­(2,21) = 0.38, *p* = 0.6871, η^2^ = 0.03511]) (Figure 1D) and falling latency ([F­(2,21) = 0.95, *p* = 0.4014, η^2^ = 0.08327]) (Figure 1E) values of mice recorded in the activity meter and the Rota-rod tests are demonstrated in Figure 1.

**Figure 1:**
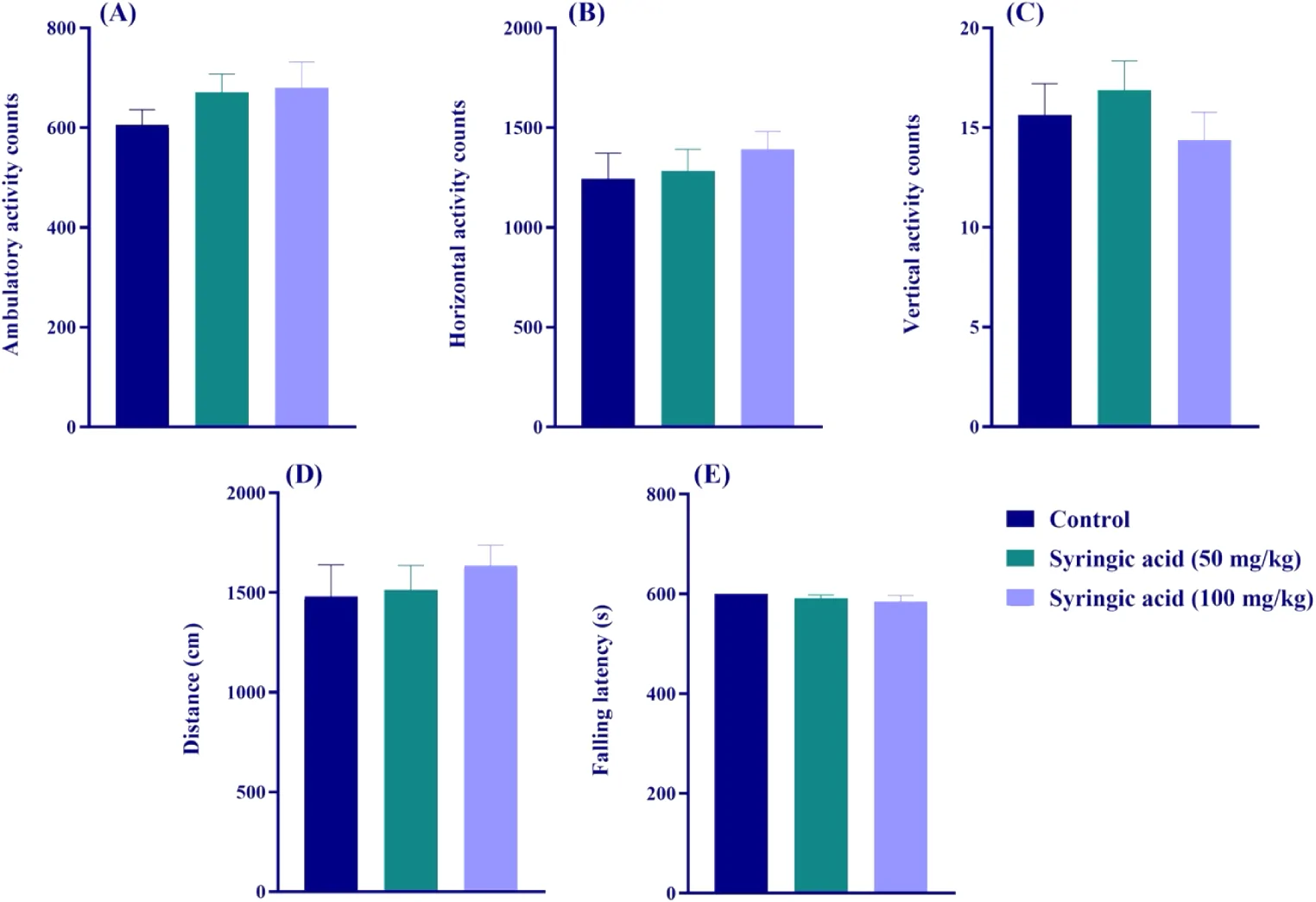
Effects of syringic acid (50 and 100 mg/kg) administrations on (A) ambulatory, (B) horizontal, and (C) vertical activity counts, together with (D) walking distance and (E) falling latency values of mice recorded in the activity meter and the Rota-rod tests. One-way ANOVA with Tukey’s HSD post hoc test, *n* = 8.

Multiple comparison analyses revealed that acute syringic acid administration (50 and 100 mg/kg) did not result in statistically significant alterations in any of these parameters.

#### 3.1.2 Findings of Tail-Clip Test

The effects of syringic acid (50 and 100 mg/kg) and reference drug (morphine, 10 mg/kg) administrations on the reaction times of mice in the tail-clip test are displayed in Figure 2A. The two-way repeated-measures ANOVA showed significant effects of treatment [F­(3,28) = 24.60, *p* <0.0001, η^2^p = 0.8504] and time [F­(3.537,99.04) = 36.99, *p*<0.0001, η^2^ *p* = 0.5692] factors on the reaction times of mice, with a significant treatment × time interaction [F­(15,140) = 25.49, *p*<0.0001, η^2^p = 0.7320].

**Figure 2:**
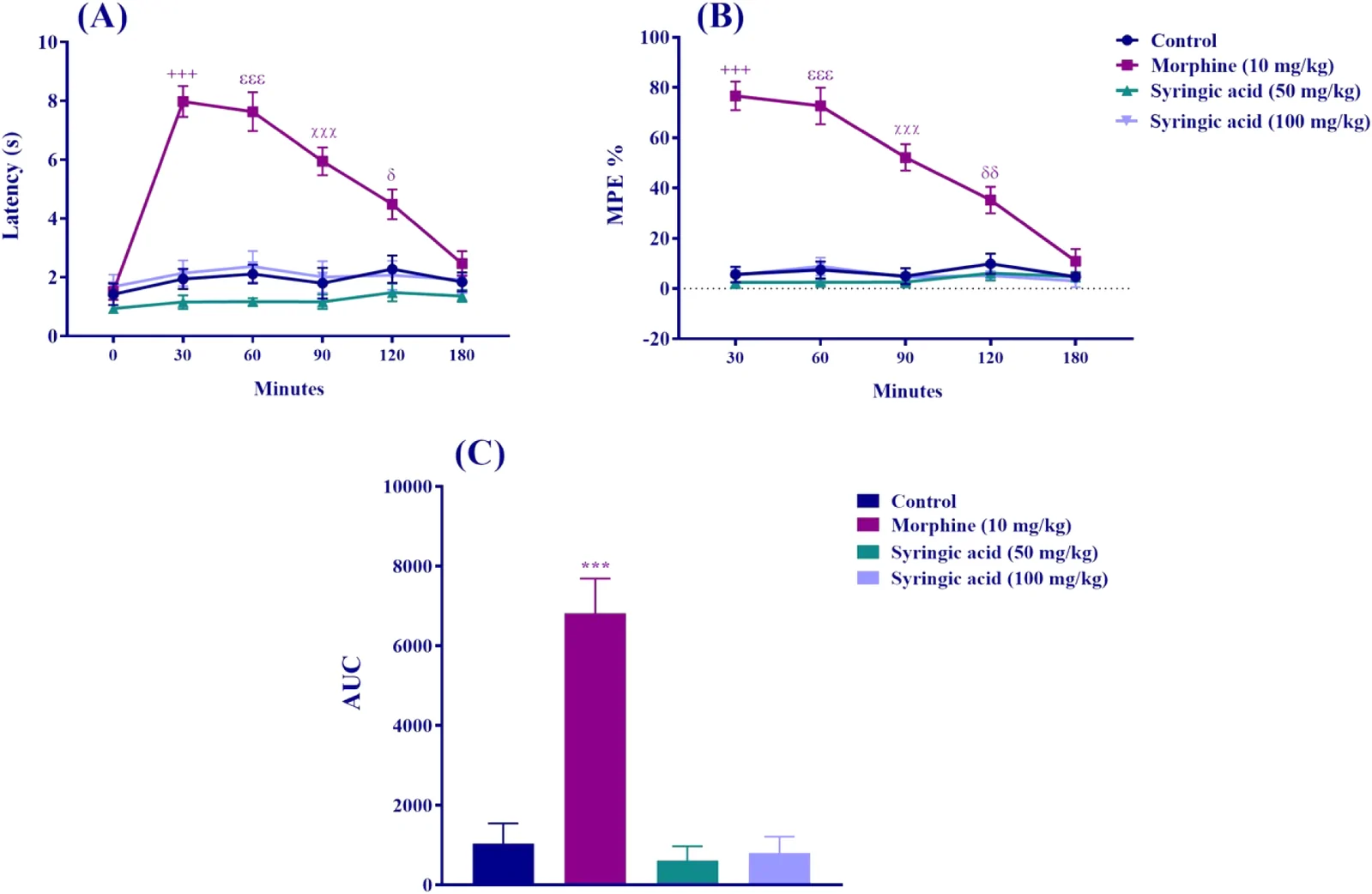
Effects of morphine (10 mg/kg) and syringic acid (50 and 100 mg/kg) administrations on the (A) response latencies, (B) MPE % values, and (C) AUC values of mice recorded in the tail-clip test. For A and B: Significance against control group at 30^th^ min ^+++^ *p*<0.001, significance against control group at 60^th^ min ^εεε^ *p*<0.001, significance against control group at 90^th^ min ^χχχ^ *p*<0.001, significance against control group at 120^th^ min ^δ^ *p*<0.05, ^δδ^ *p*<0.01. Two-way repeated-measures ANOVA with Tukey’s HSD post hoc test, *n* = 8. For C: Significance against control group ***p<0.001. One-way ANOVA with Tukey’s HSD post hoc test, *n* = 8.

Figure 2B demonstrates the effects of syringic acid (50 and 100 mg/kg) and reference drug (morphine, 10 mg/kg) on MPE % values calculated in the tail-clip test. Two-way repeated-measures ANOVA analyses revealed that treatment [F­(3,28) = 85.93, *p*< 0.0001, η^2^ *p* = 0.8666] and time [F­(3.050,85.40) = 19.33, *p*<0.0001, η^2^p = 0.4084] factors were effective on MPE % values. In addition, a significant treatment × time interaction was observed [F­(12,112) = 18.34, *p*< 0.0001, η^2^ *p* = 0.6627].

Multiple comparison analyses revealed that syringic acid did not significantly alter the response latencies of mice or calculated MPE % values at the administered doses. For response times, morphine produced significant differences at 30^th^ min (mean difference = −6.038, 95% CI: −7.888 to −4.187, *p*< 0.0001), 60^th^ min (mean difference = −5.519, 95% CI: −7.763 to −3.274, *p*< 0.0001), 90^th^ min (mean difference = −4.148, 95% CI: −6.199 to −2.096, *p* = 0.0002), and 120^th^ min (mean difference = −2.208, 95% CI: −4.206 to −0.209, *p* = 0.0285) compared to control group. Similarly, MPE% values were significantly increased at 30^th^ min (mean difference = –71.07, 95% CI: −90.76 to −51.39, *p*<0.0001), 60^th^ min (mean difference = −65.25, 95% CI: −89.81 to −40.70, *p*<0.0001), 90^th^ min (mean difference = −47.27, 95% CI: −65.52 to −29.01, *p*<0.0001), and 120^th^ min (mean difference = −25.39, 95% CI: −44.72 to −6.054, *p* = 0.0094) with respect to control group. Results regarding AUC values were also parallel with these findings [F­(3,112) = 27.30, *p*<0.0001, η^2^ = 0.4224] (Figure 2C). AUC values of morphine-treated group were significantly increased compared to control group (mean difference = −5771, 95% CI: −7890 to −3652, *p*<0.0001).

#### 3.1.3 Findings of Tail-Immersion Test

The effects of syringic acid (50 and 100 mg/kg) and reference drug (morphine, 10 mg/kg) administrations on the reaction times of mice in the tail-immersion test are exhibited in Figure 3A. The two-way repeated-measures ANOVA showed significant effects of treatment [F­(3,28) = 23.70, *p*<0.0001, η^2^ *p* = 0.7418] and time [F­(3.967,111.1) = 42.12, *p*<0.0001, η^2^ *p* = 0.6007] factors on the reaction times of mice, with a significant treatment × time interaction [F­(15,140) = 18.38, *p*<0.0001, η^2^ *p* = 0.6632].

**Figure 3:**
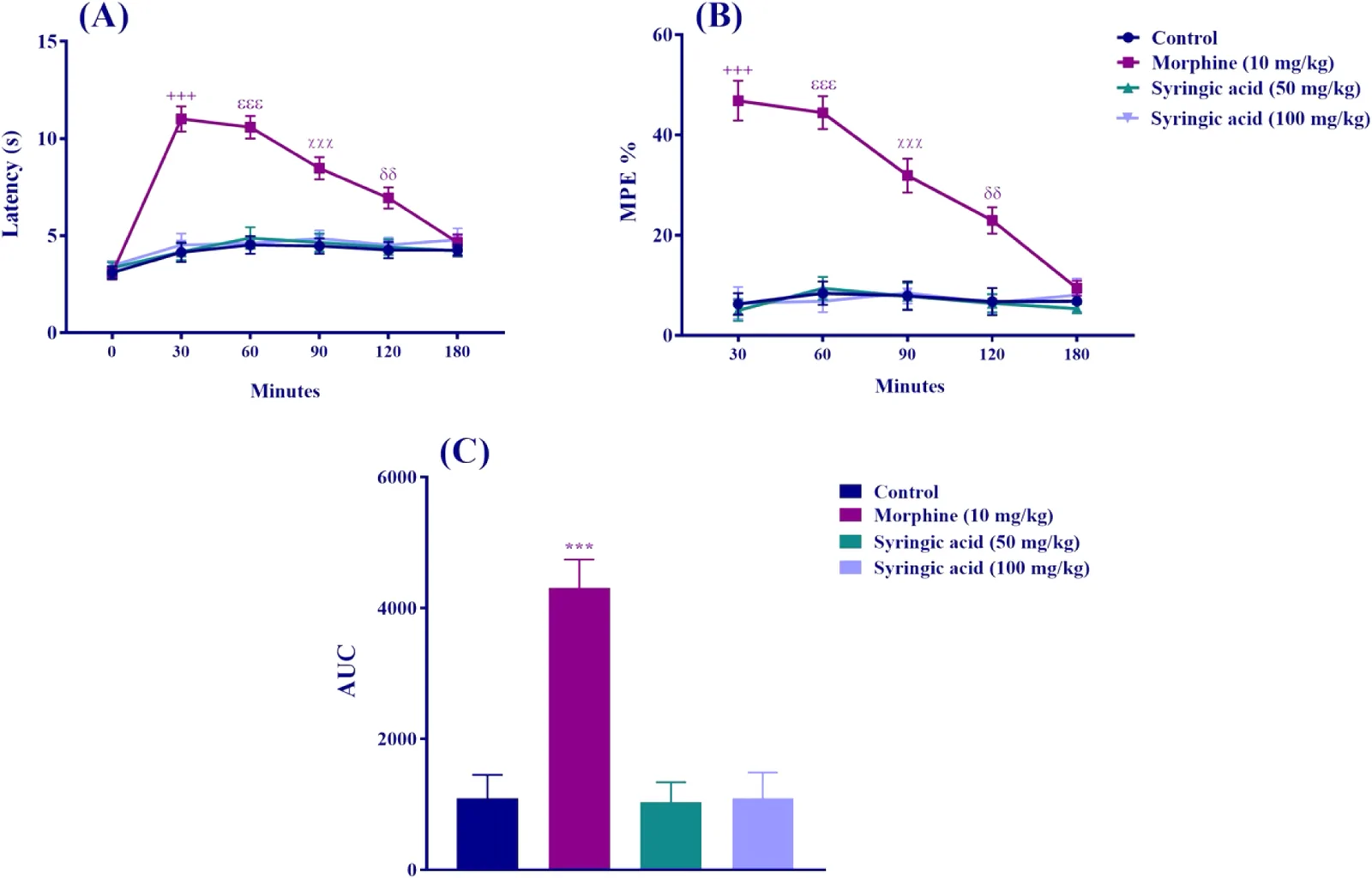
Effects of morphine (10 mg/kg) and syringic acid (50 and 100 mg/kg) administrations on the (A) response latencies, (B) MPE % values, and (C) AUC values of mice recorded in the tail-immersion test. For A and B: Significance against control group at 30^th^ min ^+++^ *p*<0.001, significance against control group at 60^th^ min ^εεε^ *p*<0.001, significance against control group at 90^th^ min ^χχχ^ *p*<0.001, significance against control group at 120^th^ min ^δδ^ *p*<0.01. Two-way repeated-measures ANOVA with Tukey’s HSD post hoc test, *n* = 8. For C: Significance against control group ***p<0.001. One-way ANOVA with Tukey’s HSD post hoc test, *n* = 8.

Figure 3B displays the effects of syringic acid (50 and 100 mg/kg) and reference drug (morphine, 10 mg/kg) on MPE % values calculated in the tail-immersion test. Two-way repeated-measures ANOVA analyses revealed that treatment [F­(3,28) = 50.14, *p*<0.0001, η^2^ *p* = 0.8136] and time [F­(3.545,99.25) = 14.88, *p*<0.0001, η^2^ *p* = 0.3470] factors were effective on MPE % values. In addition, a significant treatment × time interaction was observed [F­(12,112) = 13.53, *p*<0.0001, η^2^ *p* = 0.5918].

Multiple comparison analyses revealed that syringic acid did not significantly alter the response latencies of mice or calculated MPE % values at the administered doses. For response times, morphine produced significant differences at 30^th^ min (mean difference = −6.865, 95% CI: −9.264 to −4.466, *p* < 0.0001), 60^th^ min (mean difference = −6.068, 95% CI: −8.236 to −3.899, *p* < 0.0001), 90^th^ min (mean difference = −4.009, 95% CI: −6.067 to −1.950, *p* = 0.0004), and 120^th^ min (mean difference = −2.679, 95% CI: −4.688 to −0.6691, *p* = 0.0084) compared to control group. Similarly, MPE% values were significantly increased at 30^th^ min (mean difference = −40.56, 95% CI: −54.22 to −26.89, *p*<0.0001), 60^th^ min (mean difference = −36.03, 95% CI: −47.89 to −24.16, *p*<0.0001), 90^th^ min (mean difference = −23.98, 95% CI: −36.87 to −11.09, *p* = 0.0005), and 120^th^ min (mean difference = −16.20, 95% CI: −27.23 to −5.171, *p* = 0.0038) with respect to control group. Results regarding AUC values were also parallel with these findings [F­(3,112) = 18.48, *p* < 0.0001, η^2^ = 0.3311] (Figure 3C). AUC values of morphine-treated group were significantly increased compared to control group (mean difference = −3216, 95% CI: −4604 to −1828, *p*<0.0001).

#### 3.1.4 Findings of Hot-Plate Test

The effects of syringic acid (50 and 100 mg/kg) and reference drug (morphine, 10 mg/kg) administrations on the reaction times of mice in the hot-plate test are demonstrated in Figure 4A. The two-way repeated-measures ANOVA showed significant effects of treatment [F­(3,28) = 31.64, *p* < 0.0001, η^2^ *p* = 0.7785] and time [F­(4.030,112.8) = 21.44, *p*<0.0001, η^2^ *p* = 0.4337] factors on the reaction times of mice, with a significant treatment × time interaction [F­(15,140) = 14.11, *p*<0.0001, η^2^ *p* = 0.6018].

**Figure 4:**
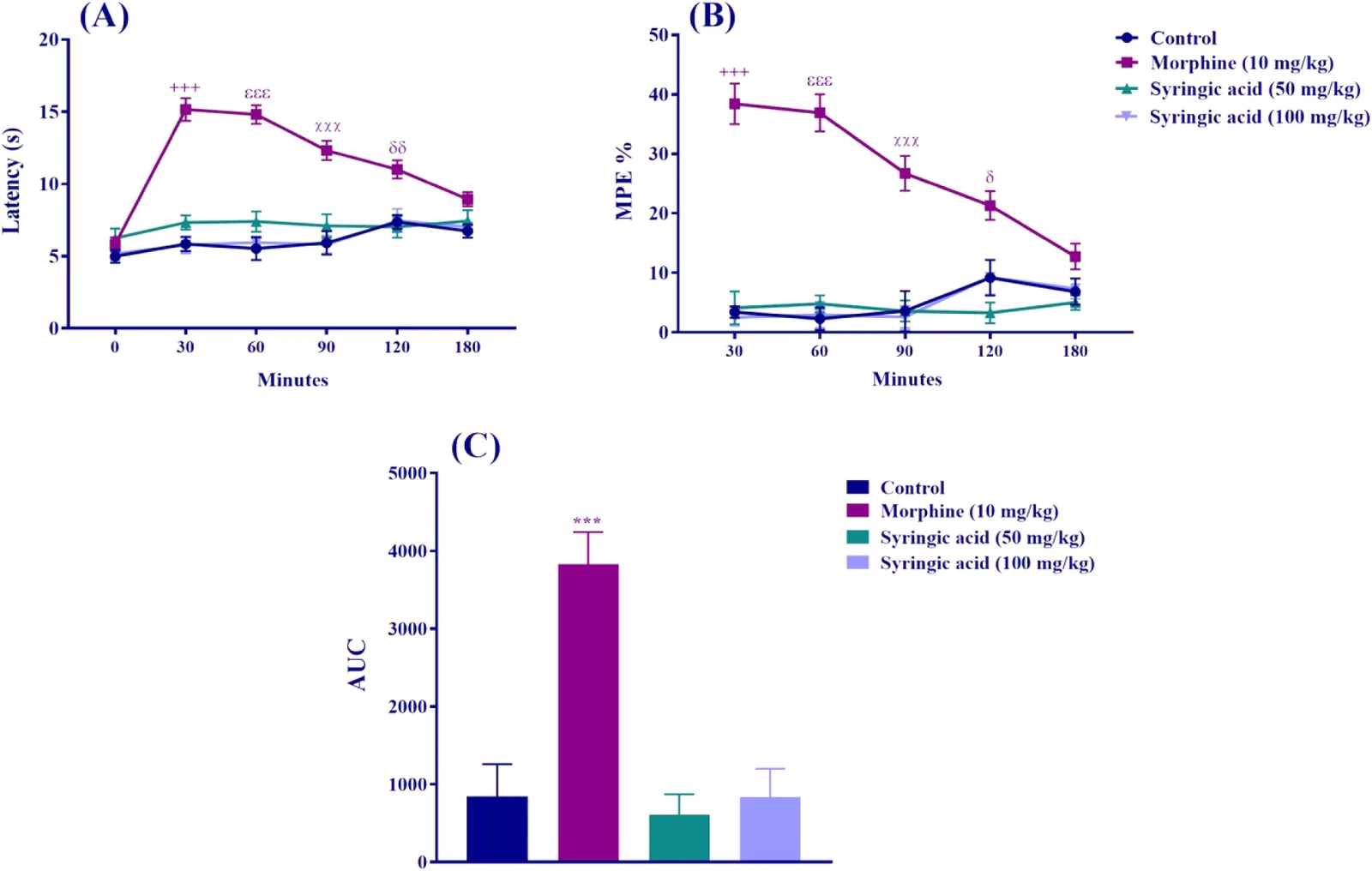
Effects of morphine (10 mg/kg) and syringic acid (50 and 100 mg/kg) administrations on the (A) response latencies, (B) MPE % values, and (C) AUC values of mice recorded in the hot-plate test. For A and B: Significance against control group at 30^th^ min ^+++^ *p*<0.001, significance against control group at 60^th^ min ^εεε^ *p*<0.001, significance against control group at 90^th^ min ^χχχ^ *p*<0.001, significance against control group at 120^th^ min ^δ^ *p*<0.05, ^δδ^ *p*<0.01. Two-way repeated measures ANOVA with Tukey’s HSD post hoc test, *n* = 8. For C: Significance against control group ***p<0.001. One-way ANOVA with Tukey’s HSD post hoc test, *n* = 8.

Figure 4B shows the effects of syringic acid (50 and 100 mg/kg) and reference drug (morphine, 10 mg/kg) on MPE % values calculated in the hot-plate test. Two-way repeated-measures ANOVA analyses revealed that treatment [F­(3,28) = 5 9.87, *p*<0.0001, η^2^ *p* = 0.8035] and time [F­(3.539,99.10) = 2.96, *p* = 0.0285, η^2^ *p* = 0.0956] factors were effective on MPE % values. In addition, a significant treatment × time interaction was observed [F­(12,112) = 9.82, *p*<0.0001, η^2^ *p* = 0.5127].

Multiple comparison analyses revealed that syringic acid did not significantly alter the response latencies of mice or calculated MPE % values at the administered doses. For response times, morphine produced significant differences at 30^th^ min (mean difference = −9.321, 95% CI: −12.07 to −6.570, *p*<0.0001), 60^th^ min (mean difference = −9.284, 95% CI: −12.26 to −6.311, *p*<0.0001), 90^th^ min (mean difference = −6.408, 95% CI: −9.481 to −3.334, *p* = 0.0002), and 120^th^ min (mean difference = −3.645, 95% CI: −5.936 to −1.354, *p* = 0.0021) compared to control group. Similarly, MPE% values were significantly increased at 30^th^ min (mean difference = −35.01, 95% CI: −46.27 to −23.76, *p*<0.0001), 60^th^ min (mean difference = –34.61, 95% CI: −45.49 to −23.73, *p*<0.0001), 90^th^ min (mean difference = −23.13, 95% CI: −36.16 to −10.11, *p* = 0.0008), and 120^th^ min (mean difference = –12.13, 95% CI: −23.42 to −0.8387, *p* = 0.0337) with respect to control group. Results regarding AUC values were also parallel with these findings [F­(3,112) = 17.31, *p*<0.0001, η^2^ = 0.3168] (Figure 4C). AUC values of morphine-treated group were significantly increased compared to control group (mean difference = −2980, 95% CI: −4340 to −1620, *p*<0.0001).

#### 3.1.5 Findings of Acetic Acid-Induced Writhing Test

The effects of acute syringic acid (50 and 100 mg/kg) and diclofenac (20 mg/kg) administrations on the writhing counts of animals in the acetic acid-induced writhing test are demonstrated in Figure 5A [F­(3,28) = 14.36, *p*<0.0001, η^2^ = 0.6061]. Analysis by the Tukey HSD test displayed that the number of writhes was significantly lower in the groups treated with 50 mg/kg (mean difference = 16.38, 95% CI: 6.668 to 26.08, *p* = 0.0004) and 100 mg/kg (mean difference = 16.63, 95% CI: 6.918 to 26.33, *p* = 0.0004) doses of syringic acid and the reference drug diclofenac (mean difference = 22.00, 95% CI: 12.29 to 31.71, *p*<0.0001) compared to control group. The antinociceptive percentages of syringic acid and diclofenac in the acetic acid-induced writhing test are shown in Table 1.

**Figure 5:**
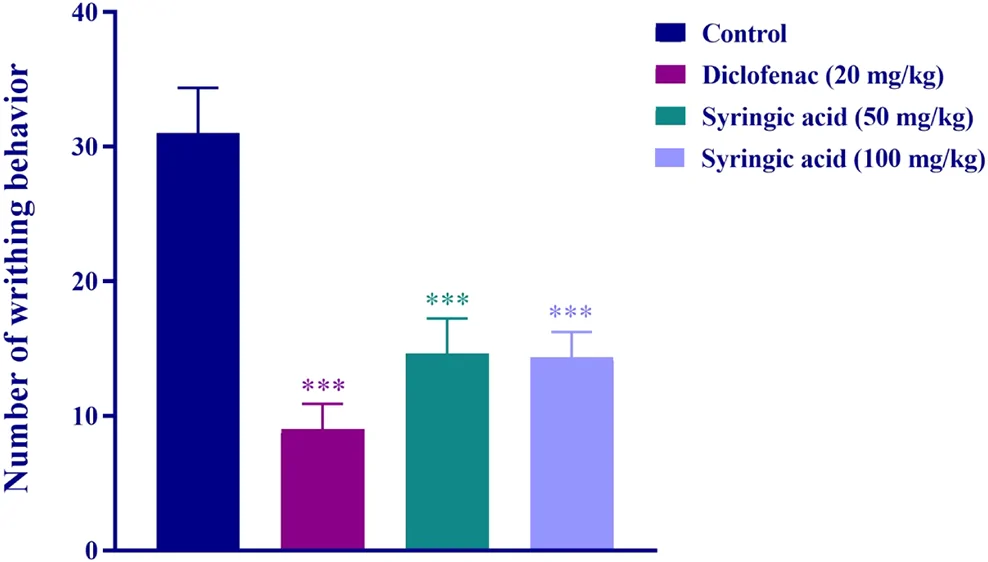
Effects of diclofenac (20 mg/kg) and syringic acid (50 and 100 mg/kg) administrations on the number of writhing behaviors of mice recorded in the acetic acid-induced writhing test. Significance against control group ***p<0.001. One-way ANOVA with Tukey’s HSD post hoc test, *n* = 8.

**Table 1:** Antinociceptive Percentages of Syringic Acid (50 and 100 mg/kg) and Diclofenac (20 mg/kg) in the Acetic Acid-Induced Writhing Test

	Antinociceptive %
Diclofenac	70.97
Syringic acid-50	52.82
Syringic acid-100	53.63

#### 3.1.6 Findings of Formalin Test

Figure 6A displays the effects of syringic acid (50 and 100 mg/kg) and reference drug (morphine, 10 mg/kg) on the paw-licking times of mice in the early stage of formalin test [F­(3,28) = 27.20, *p*<0.0001, η^2^ = 0.7445]. Multiple comparison analyses revealed that acute syringic acid administration (50 and 100 mg/kg) did not significantly change the paw-licking time of animals in the first stage of the test. The reference drug, morphine, significantly reduced the paw-licking times of animals at this phase of the test with respect to control group (mean difference = 7 7.00, 95% CI: 50.93 to 103.1, *p*<0.0001).

**Figure 6:**
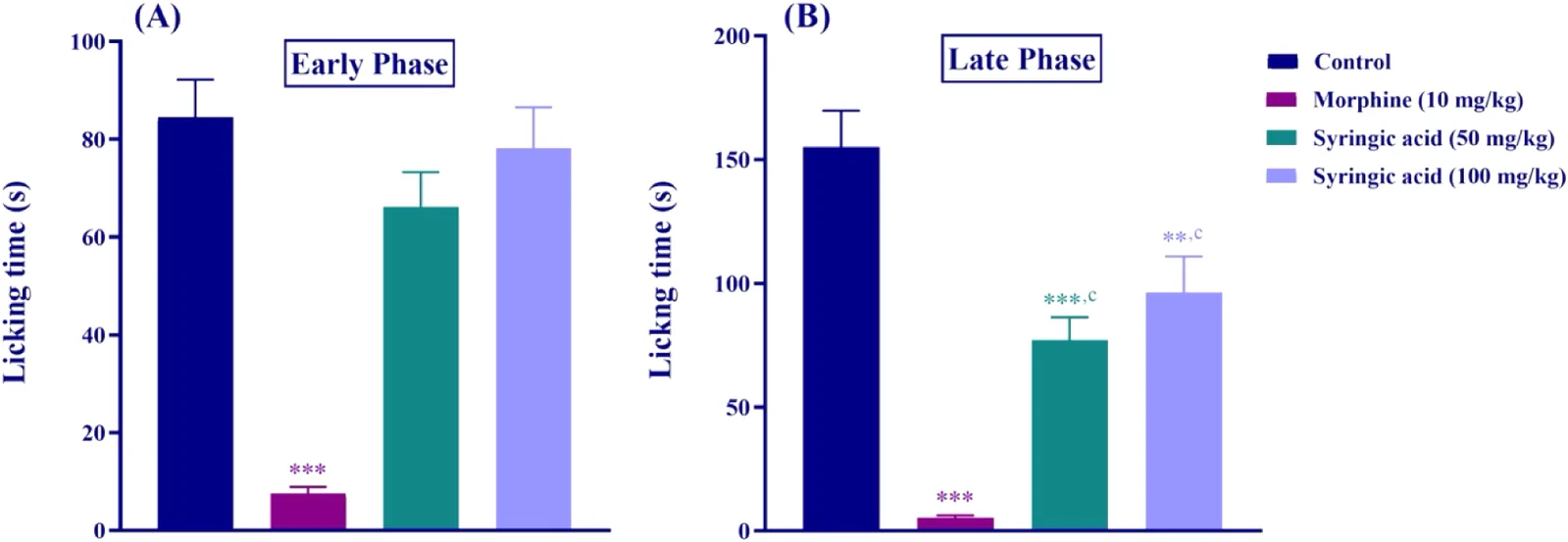
Effects of morphine (10 mg/kg) and syringic acid (50 and 100 mg/kg) administrations on the licking time of mice recorded in the (A) early and (B) late stage of formalin test. Significance against control group **p<0.01, ***p<0.001; Significance against morphine group ^c^ *p*<0.001. One-way ANOVA with Tukey’s HSD post hoc test, *n* = 8.

The effects of syringic acid (50 and 100 mg/kg) and reference drug (morphine, 10 mg/kg) administrations on the paw licking times of mice in the late stage of formalin test are exhibited in Figure 6B [F­(3,28) = 28.98, *p*<0.0001, η^2^ = 0.7564]. Tukey’s post hoc analysis indicated that syringic acid at 50 mg/kg (mean difference = 78.00, 95% CI: 33.74 to 122.3, *p* = 0.0003) and 100 mg/kg (mean difference = 58.75, 95% CI: 14.49 to 103.0, *p* = 0.0059) doses, as well as the reference drug morphine (mean difference = 149.6, 95% CI: 105.4 to 193.9, *p*<0.0001), significantly reduced the late phase paw-licking duration of mice relative to the control group. Table 2 exhibits the antinociceptive percentages of syringic acid and morphine in both phases of this test.

**Table 2:** Antinociceptive Percentages of Syringic Acid (50 and 100 mg/kg) and Reference Drug (Morphine, 10 mg/kg) in the Phases of Formalin Test

	Antinociceptive % (early phase)	Antinociceptive % (late phase)
Morphine	91.12	96.53
Syringic acid-50	21.75	50.32
Syringic acid-100	7.54	37.90

#### 3.1.7 Findings of Carrageenan-Induced Inflammation Test

Figure 7 illustrates the effects of syringic acid (50 and 100 mg/kg) and diclofenac (20 mg/kg) on the percentage paw swelling values of rats, evaluated at 1 h [F­(3,28) = 3.19, *p* = 0.0387, η^2^ = 0.2550], 2 h [F­(3,28) = 8.36, *p* = 0.0004, η^2^ = 0.4724], 3 h [F­(3,28) = 8.86, *p* = 0.0003, η^2^ = 0.4869], 4 h [F­(3,28) = 7.69, *p* = 0.0007, η^2^ = 0.4519], and 24 h [F­(3,28) = 0.95, *p* = 0.4302, η^2^ = 0.09232] after carrageenan injection.

**Figure 7:**
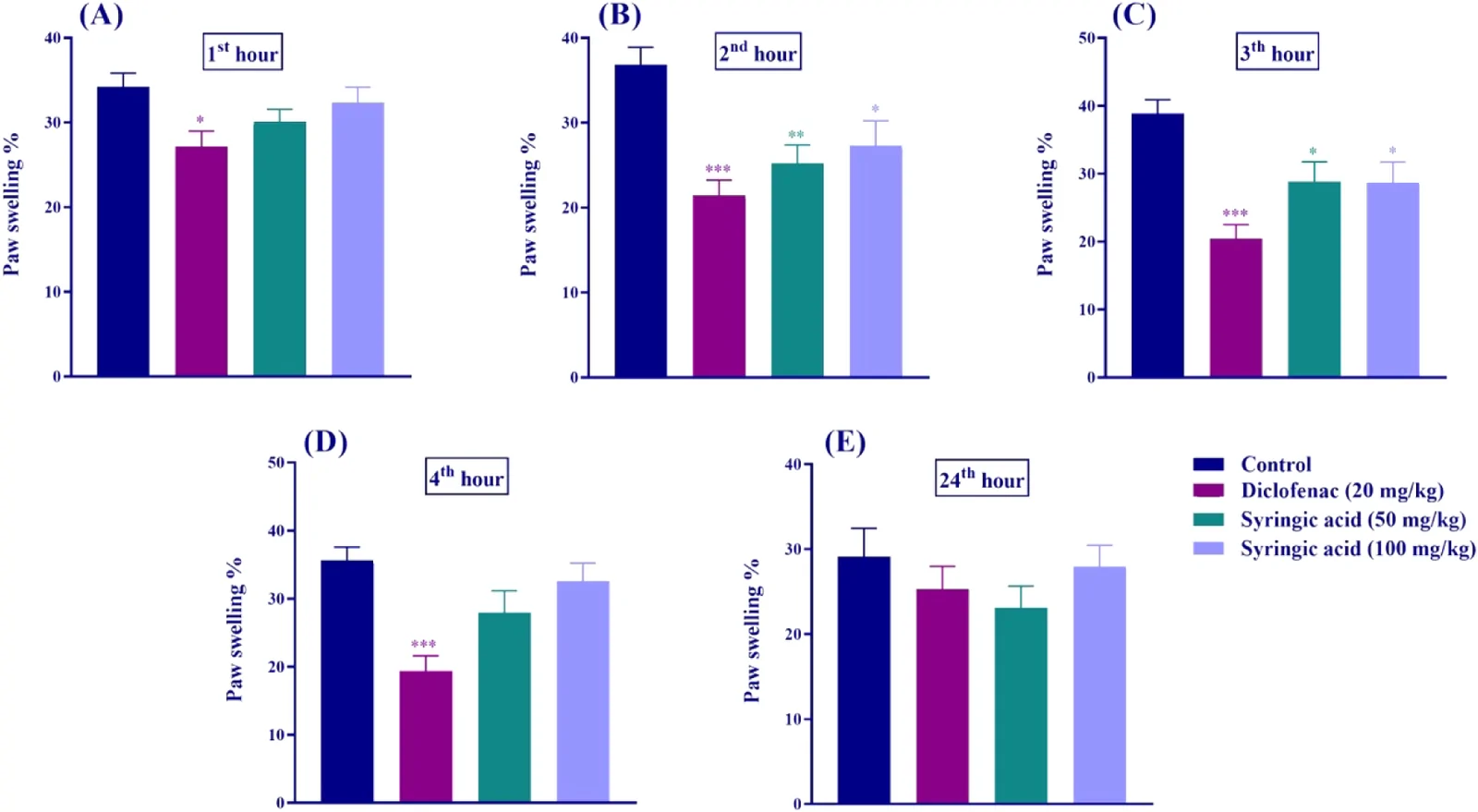
Effects of diclofenac (20 mg/kg) and syringic acid (50 and 100 mg/kg) administrations on the percentage paw swelling values of rats (A) 1 h, (B) 2 h, (C) 3 h, (D) 4 h, and (E) 24 h after carrageenan injection in carrageenan-induced inflammation test. Significance against control group *p < 0.05, **p<0.01, ***p<0.001. One-way ANOVA with Tukey’s HSD post hoc test, *n* = 8.

According to multiple comparison analyses, diclofenac (the reference drug) significantly reduced paw edema in rats at 1 h (mean difference = 7.087, 95% CI: 0.4928 to 13.68, *p* = 0.0316), 2 h (mean difference = 15.43, 95% CI: 6.653 to 24.20, *p* = 0.0003), 3 h (mean difference = 18.47 95% CI: 8.664 to 28.27, *p* = 0.0001), and 4 h (mean difference = 16.21, 95% CI: 6.368 to 26.05, *p* = 0.0006) after carrageenan injection compared to the control group, whereas syringic acid was effective only at 2 h (for 50 mg/kg mean difference = 11.63, 95% CI: 2.855 to 20.40, *p* = 0.0060; for 100 mg/kg mean difference = 9.578, 95% CI: 0.8037 to 18.35, *p* = 0.0284) and 3 h (for 50 mg/kg mean difference = 10.05, 95% CI: 0.2489 to 19.86, *p* = 0.0429; for 100 mg/kg mean difference = 10.17, 95% CI: 0.3693 to 19.98, *p* = 0.0398). However, 24 h after carrageenan injection, neither syringic acid nor diclofenac, at the administered doses, induced a significant alteration in the paw edema.

#### 3.1.8 Findings of Gastric Ulceration Studies

The effects of acute syringic acid (50 and 100 mg/kg) and diclofenac (20 mg/kg) administrations on the ulcer scores of mice are demonstrated in Figure 8 [F­(3,28) = 27.95, *p*<0.0001, η^2^ = 0.7496]. Multiple comparison analyses revealed that syringic acid did not produce any macroscopically detectable gastric lesions or ulcers. There was a significant increase in the ulcer scores of the mice after the administration of the reference drug diclofenac relative to the control group (mean difference = −3.500, 95% CI: −4.696 to −2.304, *p*<0.0001).

**Figure 8:**
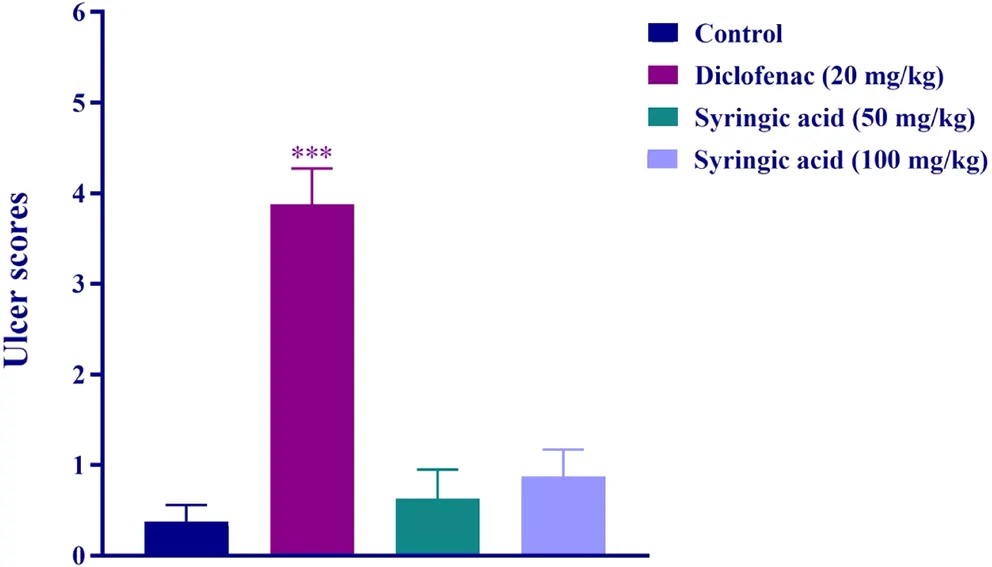
Effects of diclofenac (20 mg/kg) and syringic acid (50 and 100 mg/kg) administrations on the ulcer scores of mice. Significance against control group ^***^ *p*<0.001. One-way ANOVA with Tukey’s HSD post hoc test, *n* = 8.

### 3.2 *In Vitro* Enzyme Studies

The IC_50_ values of syringic acid on COX-1, COX-2, and 5-LOX were calculated as described in the Methods section and are presented in Table 3 and Figure 9.

**Table 3:** IC_50_ Values on the Enzymes (μM)^a^

	COX-1	COX-2	5-LOX
Syringic acid	386 (95% CI: 217.6 to 756.1)	0.77 (95% CI: 0.3862 to 1.467)	7.21 (95% CI: 3.93 to 11.15)
SC560	0.009949 (95% CI: 0.007025 to 0.01378)	nt	nt
Celecoxib	nt	0.7347 (95% CI: 0.587 to 0.912)	nt
NDGA	nt	nt	2.84 (95% CI: 1.93 to 4.347)

a nt, not tested.

**Figure 9:**
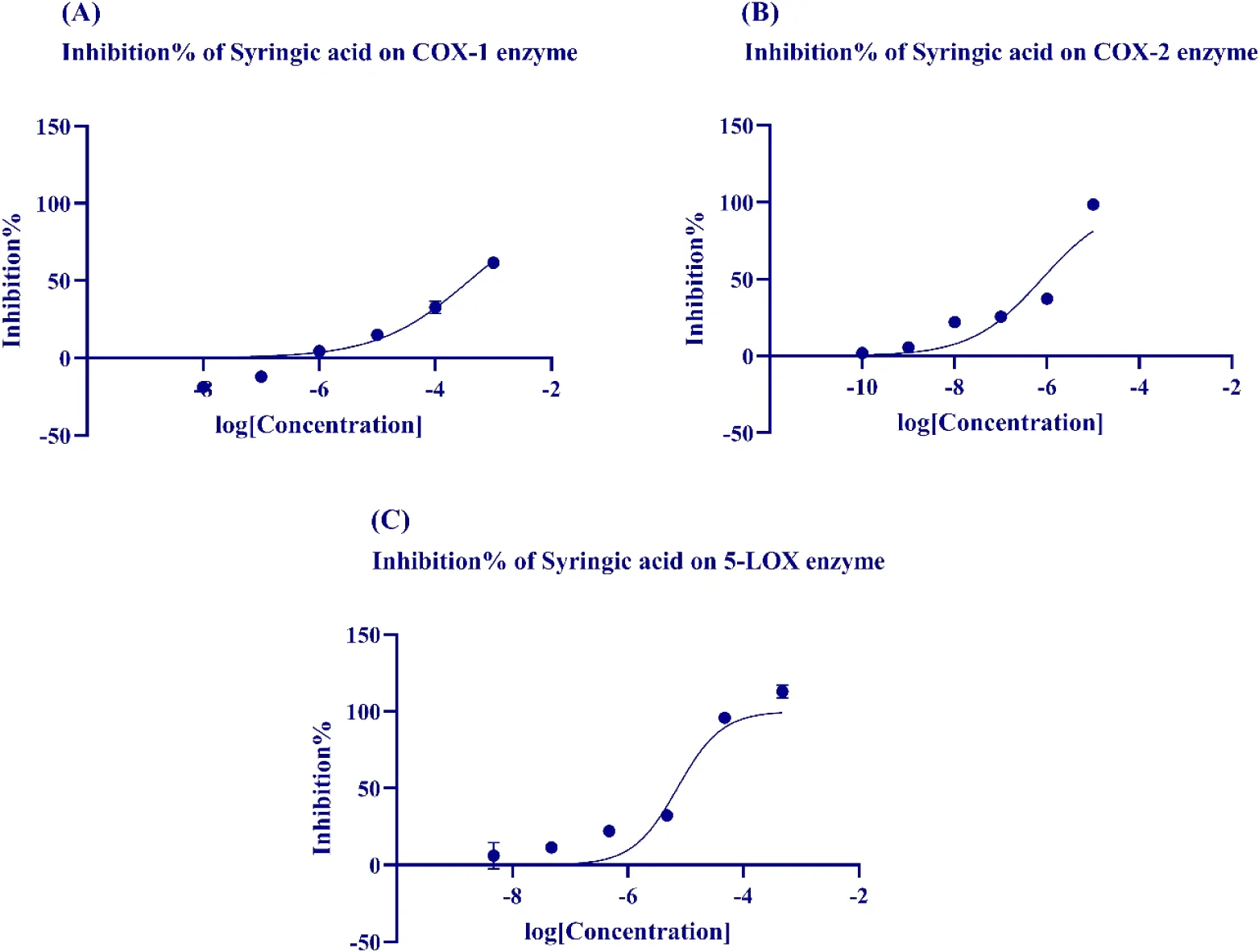
Effects of syringic acid on (**A**) COX-1, (**B**) COX-2 and (**C**) 5-LOX enzyme.

IC_50_ value of SC560 was calculated 0.009949 μM (95% CI: 0.007025 to 0.01378) while SA inhibited COX-1 enzyme activity at 386 μM (95% CI: 217.6 to 756.1). It’s IC_50_ value (0.77 μM, 95% CI: 0.3862 to 1.467) against COX-2 is very close to celecoxib (0.7347 μM, 95% CI: 0.587 to 0.912). And its IC_50_ value on 5-LOX is calculated as 7.21 μM (95% CI: 3.93 to 11.15) while NDGA inhibited 50% of the 5-LOX activity at 2.84 μM (95% CI: 1.93 to 4.347).

### 3.3 *In Silico* Studies

#### 3.3.1 Results of Molecular Docking Studies

The two-dimensional (2D) and three-dimensional (3D) relationships between syringic acid and the COX-1 enzyme are presented in Figure 10A. Molecular docking analysis revealed that syringic acid was docked into the COX-1 binding site but is located more than 4 Å away from key residues Tyr355 and Arg120, which are critical for COX inhibition,^48,49^ and does not interact with the enzyme. Thus, the syringic acid–COX-1 complex is thought to be unstable, and therefore this docking pose may represent a false or nonproductive binding mode. Overall, these results indicate that syringic acid is unlikely to act as a COX-1 inhibitor.

**Figure 10:**
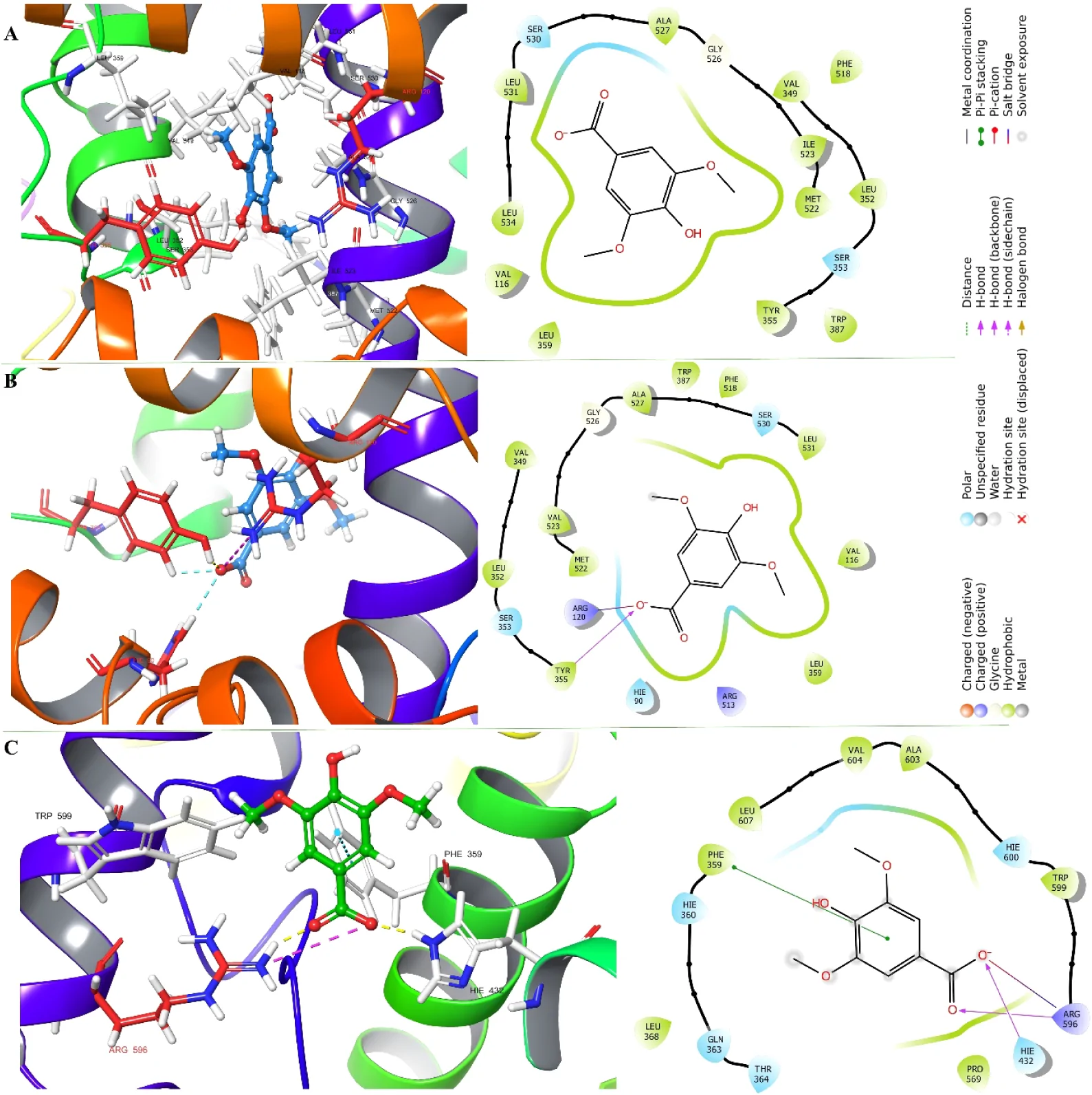
2D and 3D poses of syringic acid at active pockets of (**A**) COX-1, (**B**) COX-2, and (**C**) 5-LOX enzymes. In both 3D poses, the carbons of the key residues for the enzyme inhibitory activity are red, the carbons of the other interacting residues are white.

Figure 10B displays the 2D and 3D relationships between syringic acid and the COX-2 enzyme. Molecular docking analysis revealed that syringic acid forms one H-bond with Tyr355, two aromatic H-bonds with Arg120 and His90, and a salt bridge with Arg120. In other words, syringic acid interacts with Arg120 and Tyr355, which are required for the COX-2 enzyme,^48,49^ as mentioned above. Furthermore, this phenolic compound also interacts with His90, located in the additional pocket of COX-2, which is one of the important amino acids in the COX-2 selectivity. On the basis of these results, it can be suggested that syringic acid, which interacts with all these important amino acids, can inhibit the COX-2 enzyme by binding to it.

2D and 3D poses of syringic acid in the active pocket of the 5-LOX enzyme are demonstrated in Figure 10C. Molecular docking analysis revealed that syringic acid also forms two H-bonds with His432 and Arg596, a salt bridge with Arg596, and a π–π stacking with Phe359. In previous studies, Arg596 was described as the essential amino acid for the LOX inhibitory activity.^50^ Therefore, it can be concluded that syringic acid docked with 5-LOX enzyme. On the other hand, it is possible that interactions with other active pocket amino acids (Phe359 and His432) induced inhibitory activity and/or stabilized the ligand-protein complex.

#### 3.3.2 Results of MD Simulation Studies

In MD simulation studies, the stability of the ligand-protein complex is essential for evaluating ligand-protein interactions. When the stability graphs of syringic acid-COX-2 (Figure 11A1–C1) and syringic acid-5-LOX (Figure 11A2–C2) enzyme complexes were examined, it was revealed that the Rg plot showed low fluctuations, the RMSD values of the protein were between 1–3 Å, and the fluctuation values of the interacting residues in the RMSF plot decreased. Since these obtained values are in accordance with the standards stated in the literature,^46,51^ it can be concluded that both ligand-protein complexes are stable.

**Figure 11:**
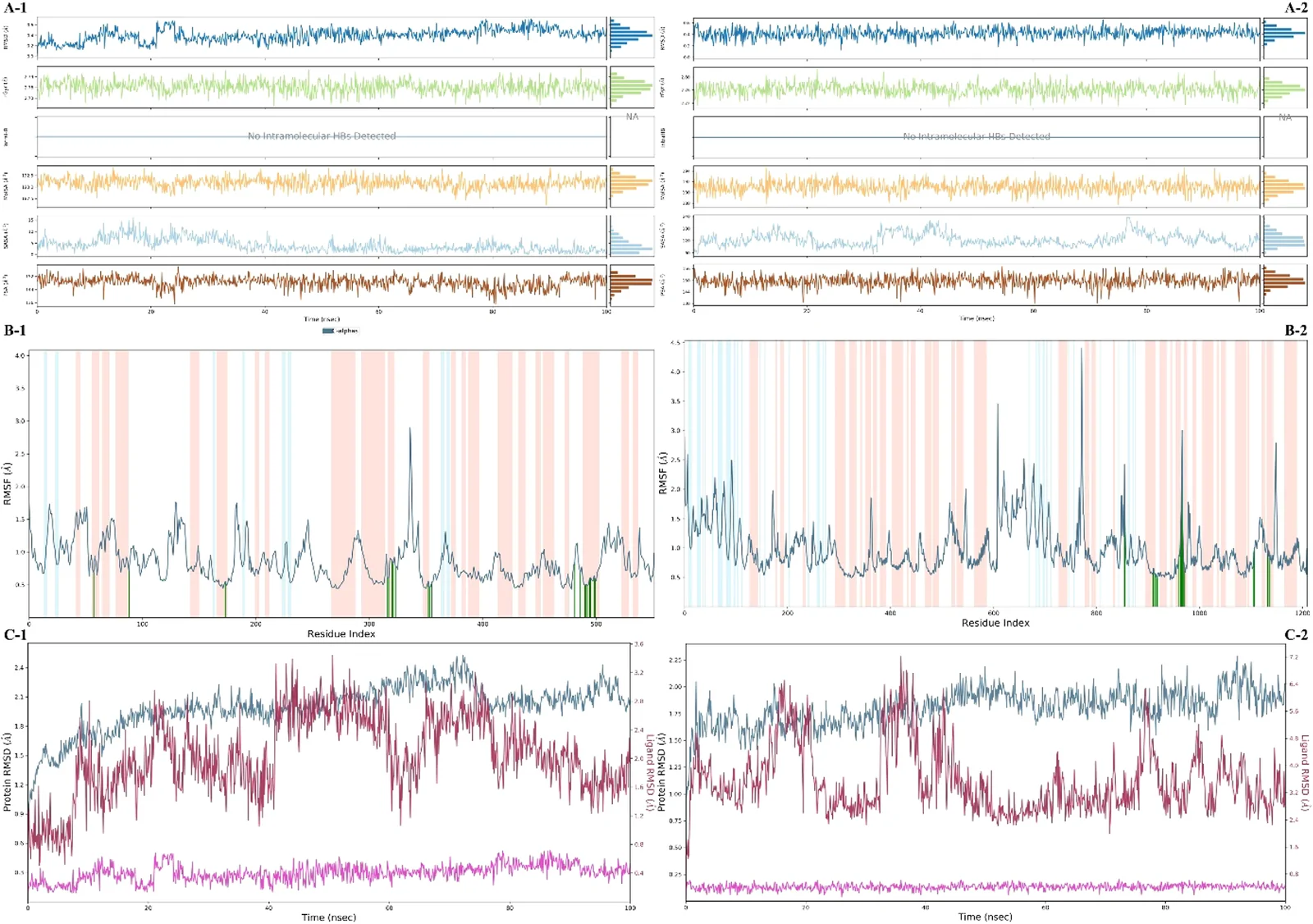
Stability diagrams of syringic acid-COX-2 (row 1) and -LOX-5 (row 2) enzyme complexes A: Ligand properties during simulation; B: RMSF plots; C: RMSD plots of Cα amino acids, ligand on protein and ligand on ligand.

It is known that for the data obtained in MD simulation studies to be reliable, the simulation must demonstrate stability throughout part or all of the simulation.^39,52^ In this study, the data obtained in the simulations for COX-2 and 5-LOX enzymes can be considered reliable because the stability was maintained throughout the simulation periods.

Figure 12 shows the interaction results of syringic acid-COX-2 enzyme complex in MD simulation studies. As mentioned above, the essential amino acids for interaction with the COX enzyme are known to be Arg120 and Tyr355. In our study, the two oxygen atoms of the carboxylic acid moiety of syringic acid formed two H-bonds with Arg120 and interacted with Tyr355 through a water-mediated H-bond. Additionally, there were some aromatic H-bonds with Tyr385, Trp387, Val523, and Ser530. This carboxylic acid was also bound to the amino acids Leu352, Ser530, and Arg513 via water-mediated H-bonds. In addition, the phenolic hydroxy group of syringic acid interacted with Tyr385 and Ser530 via H-bonding. Furthermore, one of the methoxy groups of syringic acid had the same interactions with the phenolic hydroxy group. A persistent interaction was observed between Arg120, which has the highest interaction fraction, and syringic acid. On the other hand, only two types of interactions, namely H-bonding and π–π interaction, were observed throughout the simulation. After Arg120 residue, interaction fractions above 0.5 were detected with the amino acids Tyr385, Ser530, Arg513, and Leu352, respectively. Although Tyr385 is known as a hydrophobic amino acid, direct interactions with this amino acid occurred at over 60% H-bond strength. As a result, interactions with Arg120, Leu352, Tyr355, Tyr385, Ser530, and Arg513 have an important role in the inhibitory activity of syringic acid. Although an interaction with amino acid His90 was observed as a result of docking, this interaction was determined to disappear within around 10 ns in the MDS. However, selective inhibition of COX-2 was still present due to the ligand’s interactions with Arg513, and these interactions continued to be observed with high bond strength (water-mediated H-bonding, 56%) until the end of the simulation.

**Figure 12:**
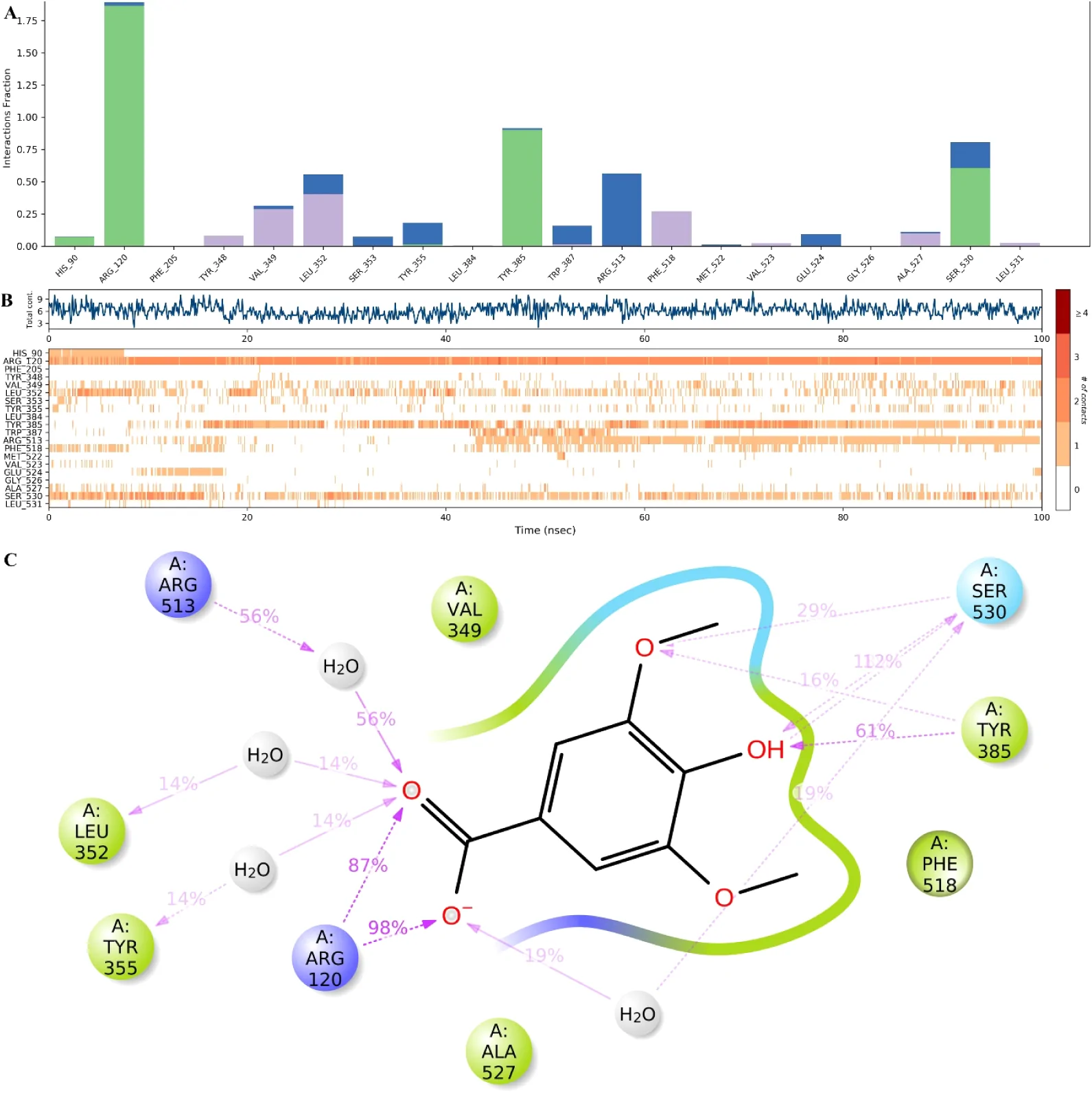
Interaction diagrams of syringic acid-COX-2 enzyme complex A: The plot of interaction fractions-interaction types by residues; B: Total interaction for residue-time plot, C: 2D pose of the contact strength of syringic acid at the active pocket of COX-2 enzyme (cut off = 10%). *In A section, green is for direct H-bond, blue is for water-mediated H-bond, and pink is for hydrophobic interactions.

Interaction results of the syringic acid-5-LOX enzyme complex in MD simulation studies are displayed in Figure 13. H bonds, salt bridges, π–π stackings and π-cations were observed during the simulation. Interactions with Arg596 were never interrupted, were seen as H-bond, water-mediated H-bond, and ionic interactions; and its interaction fraction was determined around 1.75. The H-bond strengths were calculated as 72% and 80%. On the other hand, the interaction fractions of Thr364, His432, and Trp599 amino acids were calculated to be above 0.5 and were found to be important for the interactions observed in this study. Aromatic H-bonds were also observed with the His360, His432, and Trp599 amino acids.

**Figure 13:**
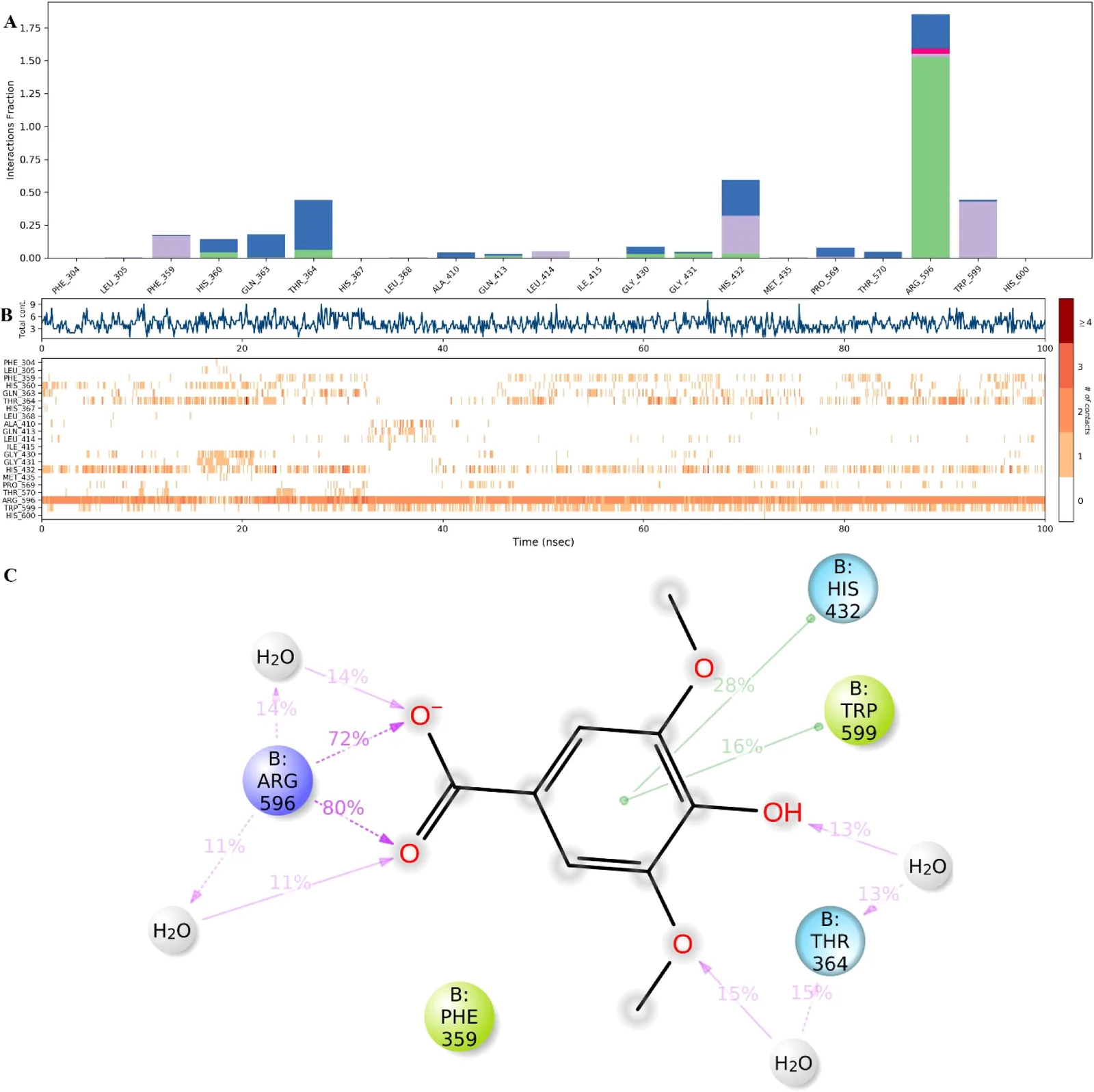
Interaction diagrams of syringic acid-5-LOX enzyme complex A: The plot of interaction fractions-interaction types by residues; B: Total interaction for residue-time plot, C: 2D pose of the contact strength of syringic acid at the active pocket of 5-LOX enzyme (cut off = 10%). *In A section, green is for direct H-bond, is blue for water-mediated H-bond, pink is for hydrophobic interactions, and red is for ionic interactions.

Syringic acid not only docked into the active site via the amino acid Arg596, which is important for inhibitory activity, but also interacted with other amino acids. However, it can be suggested that the activity is mainly related to the arginine amino acid and that other amino acids may contribute to the inhibitory activity by ensuring that the complex remains stable. The interactions occurred with Thr364 via H-bond and water-mediated H-bond; with His422 via H-bond, water-mediated H-bond, and hydrophobic interactions; and with Trp599 via water-mediated H-bond and hydrophobic interactions.

## 4 Discussion

In the present study, the antinociceptive and anti-inflammatory potentials of syringic acid were examined using various *in vivo* methods. Since agents that impair motor performance in rodents may lead to false-positive outcomes in pain assays,^27,53^ the experiments commenced with an assessment of the possible effects of syringic acid on motor activity. Data from motor activity tests showed that syringic acid, at the administered doses, did not cause statistically significant changes in ambulatory activities, vertical and horizontal activity counts, walking distances, or falling latencies in mice (Figure 1). These findings indicate that the pharmacological activities demonstrated in our study were specific.

Several reports in the literature have examined the effects of syringic acid on the motor performance of the experimental animals. For instance, Dalmagro and colleagues reported that orally administered syringic acid at doses of 0.1, 1, 10, and 100 mg/kg did not alter the number of horizontal or vertical movements of mice in the open field test.^9,10^ Comparable findings were obtained in studies in which syringic acid was administered over longer periods. Oral administration of this phytochemical to rats (20 mg/kg, 30 days) did not produce statistically significant changes in motor performance parameters evaluated by activity meter and Rota-rod devices.^17^ Likewise, Rashedinia et al. (2020) reported that oral administration of syringic acid at 100 mg/kg for 6 weeks did not alter the falling latency of rats in the Rota-rod assessments.^14^ Collectively, these studies support the findings obtained from the activity meter test in the present study.

Tail-clip and tail-immersion tests are widely preferred *in vivo* methods to assess the spinally mediated antinociceptive activity of agents. In the tail-clip test, animals respond to a noxious mechanical stimulus,^54^ whereas in the tail-immersion test, they respond to a noxious thermal stimulus applied to the tail.^28^ In this study, syringic acid did not produce any significant changes in reaction times, MPE %, or AUC values in either test (Figures 2–3), indicating that this phenolic compound does not exhibit antinociceptive activity on spinal nociceptive pathways transmitting mechanical or thermal stimuli.

Hot-plate experiments were employed to assess the potential antinociceptive effects of syringic acid against thermal nociceptive stimuli at the supraspinal level.^54^ Syringic acid did not produce significant changes in reaction times, MPE %, or AUC values at the tested doses (Figure 4). These findings demonstrate that syringic acid does not affect the supraspinal nociceptive pathways that carry thermal painful stimuli as well as the spinal nociceptive pathways.

The aceticacid-induced writhing test is frequently preferred in peripheral antinociceptive effect screening studies. Intraperitoneal injection of acetic acid triggers a viscerosomatic reflex characterized by abdominal constriction. This nociceptive response arises either through the direct activation of nonselective cation channels located in primary afferent pathways or indirectly through the stimulation of the release of several endogenous mediators (e.g., serotonin, bradykinin, prostaglandins, histamine, cytokines), as well as enhancing COX and LOX production in peripheral tissues. Inflammatory mediators released following acetic acid administration have been reported to enhance protein leakage, vascular permeability, and neutrophil infiltration in the peritoneal cavity, thereby exacerbating local inflammation.^28,54–57^ In this study, syringic acid significantly decreased the number of aceticacid-induced writhing responses in animals at the tested doses (Figure 5). All of the findings demonstrate that syringic acid exerts a peripheral antinociceptive effect. This effect may be associated with inhibition of endogenous nociceptive mediator release, suppressive activity at nerve endings of the primary afferent neurons, or inhibition of nociceptive transmission entering the dorsal horn.

In this study, the formalin test, which is commonly employed to evaluate both the central and peripheral antinociceptive effects of agents, was also conducted. Intraplantar injection of formalin in animals causes a biphasic nociceptive response. The first phase, which begins immediately following formalin administration, is termed neurogenic or early phase, whereas the second represents the inflammatory phase. Licking behavior in the early phase results from the direct activation of nociceptors in the paw and reflects the centrally mediated pain. The inflammatory phase is elicited by the release of various mediators, including histamine, prostaglandins, serotonin, and bradykinin, in peripheral tissues as well as functional alterations within the spinal dorsal horn. Both phases of the test are inhibited by centrally acting analgesics, whereas peripherally acting agents selectively suppress the late phase.^28,29,58,59^ In the present study, syringic acid inhibited the second phase of the formalin test without altering the paw-licking duration of animals in the first phase (Figure 6). These findings corroborate the results of previous assays, indicating that syringic acid exerts peripheral but not central antinociceptive activity. On the other hand, definitive conclusions regarding the absence of central mechanisms need to be confirmed with more detailed pharmacokinetic and mechanistic studies.

Several studies in the literature have reported antinociceptive activity for various plant extracts containing syringic acid.^60,61^ However, research investigating the effects of pure syringic acid on pain is limited. In a study by Pawar et al. (2021), syringic acid was orally administered to rats with experimentally induced diabetic neuropathy (12.5, 25, and 50 mg/kg) for 5 weeks. The authors reported that syringic acid reduced blood glucose levels in diabetic animals, improved mechanical and thermal allodynia and hyperalgesia, and increased motor nerve conduction velocity (m/s), which is reduced in diabetic conditions.^62^ Only one study has examined the acute antinociceptive activity of syringic acid. In this study, Okur and Şakul evaluated the antinociceptive efficacy of orally administered syringic acid (10, 50, and 100 mg/kg) in Balb/c mice using hot-plate and tail-flick tests, both of which assess responses to thermal nociceptive stimuli. Experimental results indicated that syringic acid increased MPE % values at doses of 50 and 100 mg/kg in both tests, and they suggested that this effect may be mediated through nicotinic, α_2_-adrenergic, and opioidergic receptors.^26^ The findings of that study do not align with our results, as syringic acid at 50 and 100 mg/kg doses did not exhibit any central antinociceptive activity in the tests conducted herein.

In the context of this study, the anti-inflammatory effect of syringic acid was also examined in light of the outcomes obtained from the acetic acid-induced writhing and formalin tests. For this purpose, the carrageenan-induced inflammation assay was preferred, as it is a widely used *in vivo* model with high validity and reliability for assessing the anti-inflammatory activities of drugs and other agents. In this study, unlike the reference drug diclofenac, syringic acid significantly reduced carrageenan-induced inflammation only at 2 and 3 h after carrageenan injection (Figure 7). It is well known that the initial stage (0–1 h post-injection) of the carrageenan-induced inflammation test is primarily a non-phagocytic edema stage, characterized by the release of various mediators (histamine, serotonin, bradykinin, etc.). The second stage (occurring approximately 2 h post-injection), which is characterized by fast swelling, is related to the infiltration of polymorphonuclear leukocytes (particularly neutrophils) and increased prostaglandin production (especially PGE_2_), several proinflammatory cytokines (such as TNF-α, IL-1, and IL-6), COX-2, oxygen-derived free radicals, nitric oxide, and kinin-like substances.^28,37,63^ In this context, it can be suggested that syringic acid exerts its anti-inflammatory efficacy by predominantly suppressing the second phase of the inflammatory process. The transient anti-inflammatory effect observed in this study may be related to the reported rapid elimination of syringic acid;^64^ however, pharmacokinetic studies are required to confirm this possibility.

Various plant extracts containing syringic acid have been reported to show anti-inflammatory activity in several *in vitro* studies^65,66^ as well as in inflammation models such as the carrageenan-induced inflammation test.^60,61^ Additionally, the anti-inflammatory activity of plant extracts containing syringic acid has also been demonstrated in animal models of various inflammation-related diseases.^67,68^ There are also some papers in the literature reporting the *in vitro* anti-inflammatory effects of syringic acid.^69,70^ In addition, studies employing animal models of conditions such as Parkinson’s disease, Alzheimer’s disease, hepatic encephalopathy, septic shock, diabetic wounds, asthma, obesity, liver injury, and colitis, where inflammatory processes play an important role in pathogenesis, have reported that pure syringic acid exhibits anti-inflammatory activity by reducing the elevated levels/expressions of inflammatory mediators and/or pro-inflammatory cytokines under disease conditions,^8,11,15–17,71,72^ or by increasing the reduced levels of anti-inflammatory cytokines.^11,72^ Moreover, in a study conducted in rats with diabetic neuropathy, syringic acid administered at 25 and 50 mg/kg doses was reported to reduce inflammation in the sciatic nerve, whereas a 100 mg/kg dose of this phenolic compound attenuated both inflammation and demyelination.^14^ In another study by Chanda et al. (2020), the potential anti-inflammatory activity of syringic acid administered to Wistar rats at 25 and 50 mg/kg doses was evaluated with complete Freund’s adjuvant (CFA)-induced arthritis and carrageenan-induced paw edema tests. The findings indicated that syringic acid reduced paw edema and lowered IL-6 and TNF-α levels of animals in both experimental models.^25^ These reports support the findings of this study, which demonstrate the anti-inflammatory effects of syringic acid.

The analgesic and anti-inflammatory activities of nonsteroidal anti-inflammatory drugs (NSAIDs) are mediated through the inhibition of both COX-1 and COX-2, whereas their gastrointestinal adverse effects are primarily attributed to COX-1 inhibition. Selective COX-2 inhibitors are recognized to exert potent anti-inflammatory effects while producing minimal or no associated gastrointestinal adverse effects.^37^ Accordingly, this study also evaluated the potential risk of gastric ulceration associated with acute syringic acid administration. The experimental results demonstrated that the reference drug diclofenac led to an increase in ulcer scores, whereas syringic acid did not induce any increase at the administered doses (Figure 8). The present findings are in agreement with a previous report indicating that syringic acid exerts a protective effect against indomethacin-induced gastric ulceration by attenuating oxidative stress, inflammation, and apoptosis.^73^

Results from *in vivo* experiments demonstrated that syringic acid possesses peripheral antinociceptive and anti-inflammatory activities without inducing notable gastrointestinal irritation. It is noteworthy that there was no statistically significant difference in the antinociceptive and anti-inflammatory effects of syringic acid at the doses used in this study. This may be related to the ceiling effect or the limited potency of this phenolic compound. Compared to reference drugs, syringic acid, at both doses, was found to have lower antinociceptive efficacy than morphine in the formalin test, while it showed comparable efficacy to diclofenac in the acetic-acid-induced writhing and carrageenan-induced inflammation assays.

To elucidate the mechanisms underlying these effects, *in vitro* study (Figure 9) and molecular modeling analyses were performed. These analyses focused on COX and LOX enzymes, which play critical roles in mediating nociceptiveand inflammatory processes.^74,75^ Obtained *in vitro* results indicated that syringic acid showed substantial inhibitory activity against both COX-2 and 5-LOX enzymes, comparable to standard drugs. On the other hand, its inhibitory activity against COX-1 enzyme can be regarded as weak, considering that the compound exhibited an IC_50_ value at concentrations above 100 μM.

Molecular modeling studies demonstrating the binding of syringic acid to COX-2 and 5-LOX suggest that the peripheral antinociceptive and anti-inflammatory effects of this phenolic compound may be associated with its interactions with these enzymes. The molecular docking studies indicated that COX-2 selectivity is associated with interactions involving the additional pocket residues His90 and Arg513, whereas no corresponding contacts were observed with COX-1 amino acids (Figure 10). These results, showing that syringic acid does not interact with the COX-1 enzyme, are consistent with findings demonstrating that syringic acid has no ulcer-inducing effect. Moreover, MD simulation studies suggest that high affinity and sustained interactions were observed with the key amino acids Arg120 and Tyr355 in COX-2 inhibition and with the essential amino acid Arg596 in 5-LOX inhibition (Figures 11–13). Furthermore, it can be suggested that interactions with other active pocket amino acids may contribute to improved system stability and, thus, inhibitory activity.

This study has several limitations. One limitation is that the anti-inflammatory effects of syringic acid were evaluated only in terms of the COX and 5-LOX enzymes, which are key enzymes in the arachidonic acid cascade. However, previous papers have revealed that syringic acid may also modulate some pro-inflammatory markers, such as prostaglandins, iNOS, IL-1β, IL-2, IL-6, IL-8, TNF-α, MCP-1, IFN-γ, and NF-κB, as well as anti-inflammatory mediators (e.g., IL-10).^8,11,15–17,64,71,72^ Therefore, further studies are warranted to explore the possible interactions of syringic acid with these targets and to provide a more comprehensive understanding of its anti-inflammatory mechanisms. Another limitation of the present study is the lack of a detailed assessment of the pharmacokinetic profile of syringic acid. Therefore, to better elucidate the pharmacological profile of this phenolic compound, it is necessary to address various pharmacokinetic aspects such as plasma/tissue distribution and oral bioavailability, as well as pharmacodynamic details such as dose–response analysis, multiple dose treatment protocols, and determination of the ED_50_ value. An additional limitation of the study is that the ulcerogenic activity potential of syringic acid was evaluated only after acute administration, and the assessment was limited to macroscopic scoring. Therefore, the absence of gastric ulceration observed in the present study should not be generalized. More comprehensive investigations such as histopathological examination or measurement of gastric secretion following both acute and chronic administration of syringic acid are needed to better understand the effects of this phenolic compound on gastric mucosa. Finally, in this study, only male mice were used to avoid probable effects of hormonal fluctuations in females on the results. However, the efficacy of syringic acid in female mice should also be investigated to evaluate possible sex-related differences.

## 5 Conclusion

In conclusion, this study shows that syringic acid exhibits peripheral antinociceptive and anti-inflammatory activities without inducing ulcerogenic effects. The mechanism of action was further clarified by *in vitro* studies, which demonstrated that syringic acid exhibits strong inhibitory effects on COX-2 and 5-LOX enzymes while showing weak inhibition of COX-1. Furthermore, *in silico* investigations examining the interactions of this phenolic compound with COX and LOX enzymes, which are key targets closely associated with analgesia and inflammation,^74,75^ reveal that although syringic acid docked with COX-1, it did not form stable binding interactions. In contrast, syringic acid showed favorable docking profiles with the COX-2 and 5-LOX enzymes. Moreover, MD simulation results indicate that syringic acid interacts with the COX-2 and 5-LOX enzyme complexes with high affinity and sustained stability.

Recently, COX-2/5-LOX dual inhibitors have attracted considerable interest in the design of anti-inflammatory agents. This dual-inhibition strategy has been reported to enhance therapeutic efficacy while reducing the adverse effects related to selective inhibition of COX-2 and 5-LOX enzymes.^76^ In this context, syringic acid, which was demonstrated to exert dual inhibitory activity in the present study, may offer potential clinical advantages for the treatment of painful and inflammatory disorders. Nevertheless, further comprehensive studies are needed to confirm this hypothesis.

## Data Availability

Data will be made available on request. The videos can be viewed via youtu.be/LhJ1dTSbgs0 (**Video 1**) and youtu.be/l3o3IhrDBgU (**Video 2**).
